# Correlation between Radiological and Pathological Findings in Patients with *Mycoplasma pneumoniae* Pneumonia

**DOI:** 10.3389/fmicb.2016.00695

**Published:** 2016-05-11

**Authors:** Hiroshi Tanaka

**Affiliations:** NPO Sapporo Cough, Asthma, and Allergy CenterSapporo, Japan

**Keywords:** radiological–pathological correlation, open lung biopsy, CT scan, centrilobular nodes, bronchovascular bundles thickening, host cell-mediated immunity

## Abstract

Studies focused on the pathological–radiological correlation of human *Mycoplasma* (*M*) *pneumoniae* pneumonia have rarely been reported. Therefore, we extensively reviewed the literature regarding pathological and radiological studies of *Mycoplasma* pneumonia, and compared findings between open lung biopsy specimen and computed tomography (CT). Major three correlations were summarized. (1) Peribronchial and perivascular cuffing characterized by mononuclear cells infiltration was correlated with bronchovascular bundles thickening on CT, which was the most common finding of this pneumonia. (2) Cellular bronchitis in the small airways accompanied with exudates or granulation tissue in the lumen revealed as centrilobular nodules on CT. (3) Neutrophils and exudates in the alveolar lumen radiologically demonstrated as air-space consolidation or ground-glass opacities. In *M. pulmonis*-infected mice model, pathologic patterns are strikingly different according to host cell-mediated immunity (CMI) levels; treatment with interleukin-2 lead to marked cellular bronchitis in the small airways and treatment with prednisolone or cyclosporin-A lead to neutrophils and exudates in the alveolar lumen. Patients with centrilobular nodules predominant radiologic pattern have a high level of CMI, measuring by tuberculin skin test. From these findings, up-regulation of host CMI could change radiological pattern to centrilobular nodules predominant, on the other hand down-regulation of host CMI would change radiological pattern to ground-glass opacity and consolidation. It was suggested the pathological features of *M. pneumoniae* pneumonia may be altered by the level of host CMI.

## Introduction

The majority of *Mycoplasma (M) pneumoniae* respiratory infection are self-limited. An estimated 3–13% of infected persons with infection experience pneumonia, and the remains are manifested as upper respiratory tract infection [Clyde, 1993; Waites and Talkington, 2004; Walter et al., 2008; Centers for Disease Control and Prevention (CDC), 2013]. Therefore pathological specimens of human *M. pneumoniae* pneumonia are rarely obtained. Pathological descriptions of this pneumonia include marked plasma cell-rich lymphocytic infiltration in peribronchial and perivascular areas, with accumulations of macrophages, neutrophils, and lymphocytes in the alveolar spaces, foci of interstitial pneumonia, and hyperplasia of type II pneumocytes (Golden, 1944; Forsyth and Chanock, 1966; Meyers and Hirschman, 1972; Chan et al., 1999). Bronchiolitis and alveolitis with dense mononuclear cells infiltration, epithelioid cell granulation tissue filling alveolar ducts, organizing alveolar exudates, and hyaline membranes are characteristic findings in fulminant *M. pneumoniae* pneumonia (Koletsky and Weinstein, 1980; Rollins et al., 1986; Ito et al., 1995; Ebnother et al., 2001; Izumikawa et al., 2014). On the other hand, the patterns of *M. pneumoniae* pneumonia on chest radiography are non-specific segmental or lobar consolidation, bilateral diffuse reticular interstitial infiltrates (Putman et al., 1975). Computed tomography (CT) findings of this pneumonia are bronchovascular thickening, centrilobular nodules, ground-glass attenuation, or air-space consolidation (Tanaka et al., 1985; Tanaka N. et al., 1996; Reittner et al., 2000; Miyashita et al., 2009, 2014). However, there has been little report radiological–pathological correlation in human *M. pneumoniae* pneumonia (Heitzman, 1993). This review focuses on radiological–pathological correlation of *Mycoplasma* pneumonia in mice and humans, and the changes of pulmonary involvement patterns reflecting by host cell-mediated immunity (CMI) levels.

## Radiological–Pathological Correlation in Animal Models

**FIGURE 1:**
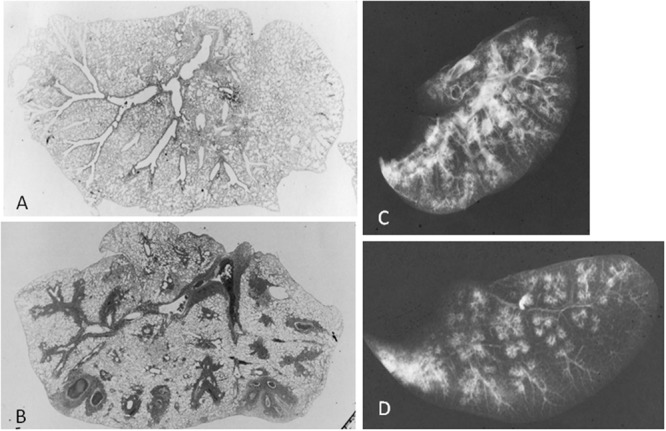
***Mycoplasma pulmonis* infected mice, 2 weeks after inoculation.** Low-magnification photomicrographs of non-infected lung **(A)** and infected lung **(B**; HE x17). **(C)** Radiograph of inflated lung of infected mice reveals bronchovascular bundles thickening, nodules, and ground-glass attenuation. **(D)** Radiograph of thin-sliced lung of infected mice shows nodules with centrilobular distribution and consolidation. Reproduced with permission from Tanaka (2016).

The pathogenesis of *Mycoplasma* infection has been studied in animal model. The pathological changes and patterns are similar to that seen in experimental infection in hamsters or mice. In **Figures 1A,B**, *M. pulmonis* inoculated mice model reveals that pathological changes consist with (1) peribronchial and perivascular mononuclear cells accumulation throughout large to small airways, (2) cellular bronchiolitis with lumen exudates and mononuclear cell in airway walls extending into adjacent alveoli (Tanaka et al., 1996a). Next, inflated lung specimens of *M. pulmonis* inoculated mice were prepared by Heitzman’s (1993) method (Markarian and Dailey, 1993). Radiological findings of the infected lungs disclosed thickening of bronchovascular bundles, centrilobular nodules, and ground-glass opacities (**Figures 1C,D**). Pathological changes clearly reflect to radiological findings in *M. pulmonis* inoculated mice.

## Immunomodulators Change the Pathological Pattern of *Mycoplasma* Pneumonia

**FIGURE 2:**
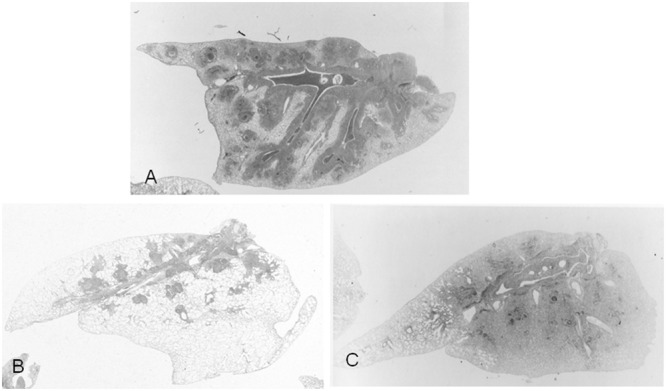
**Low-magnification photomicrographs of lung, 2 weeks after *M. pulmonis* inoculation (HE x17). (A)** Mice without treatment. **(B)** Mice treated with interleukin-2, showing marked peribronchial and perivascular lymphocyte cuffing, and no intra-alveolar inflammation. **(C)** Mice treated with PSL, disclosing predominance of intra-alveolar inflammatory cell infiltration, and a little peribronchial and perivascular lymphocyte cuffing. Reproduced with permission from Tanaka and Tamura (1989).

**FIGURE 3:**
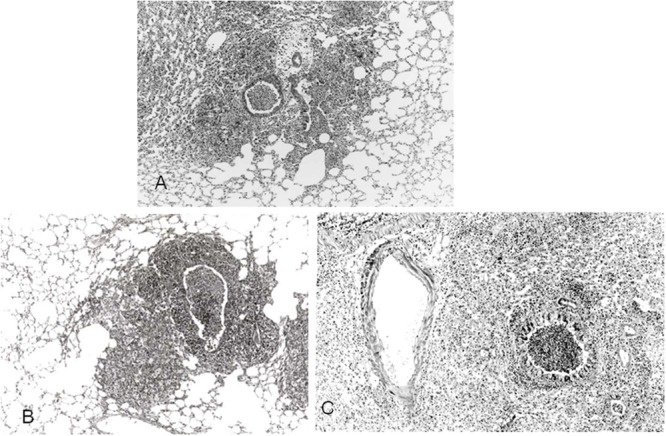
**Pathological observation of lung from *M. pulmonis* infected mice (HE x170). (A)** Mice without treatment. **(B)** Mice treated with interleukin-2, showing marked peribronchial lymphocyte cuffing and macrophage accumulation at the end bronchiole. **(C)** Mice treated with PSL, disclosing predominant intra-alveolar inflammatory cell Infiltration and faint perivascular lymphocyte cuffing. Reproduced with permission from Tanaka and Tamura (1989).

The role of T cells in the pathogenesis of *M. pneumoniae* infection can be defined by the apparent correlation of delayed-type hypersensitivity (DTH) skin reaction to *M. pneumoniae* in humans with the severity of disease (Mizutani et al., 1971). To elucidate immune-pathological mechanism of *Mycoplasma* pneumonia, the therapeutic effects of interleukin-2 (IL-2), cyclosporine A (CYA), and prednisolone (PSL) on mice model (Tanaka et al., 1996a). Mice were intra-nasally inoculated with *M. pulmonis* and were treated with IL-2, CYA, and PSL every day between Days 3 and 9, and were killed at Day 14. IL-2 is immunomodulator, especially up-regulate CMI, and CYA is immunosuppressant, especially down-regulate CMI of the host. PSL is a more powerful immunosuppressant. CMI level of the host was assessed by skin test by sheep red blood cell (SRBC). Peribronchial and perivascular mononuclear cell cuffing and accumulation of macrophages at the end of bronchiole were exacerbated in IL-2 treated mice (**Figures 2** and **3**). On the other hand, prominent intra-alveolar inflammatory cell infiltration and faint peribronchial and perivascular mononuclear cell cuffing were observed on CYA or PSL treated mice (**Figures 2** and **3**). CMI to SRBC was increased in IL-2 treated mice, however, decreased in CYA or PSL treated mice. Another *M. pneumoniae* inoculated mice model exhibited host-dependent infection-related airway obstruction and airway hyperresponsiveness associated with chemokine and T-helper type 1 pulmonary host response and not T-helper type 2 response after *M. pneumoniae* infection (Fonseca-Aten et al., 2005). Recently, the severity of the *M. pneumoniae* pneumonia seemed to depend on the host innate immunity to the *M. pneumoniae*, which might be accelerated by antecedent *M. pneumoniae* exposure (re-exposure or latent respiratory infection) through up-regulation of Toll-like receptor 2 expression on bronchial epithelial cells and alveolar macrophages using mice model (Saraya et al., 2011, 2014).

## Radiological Assessment of *M. pneumoniae* Pneumonia

**FIGURE 4:**
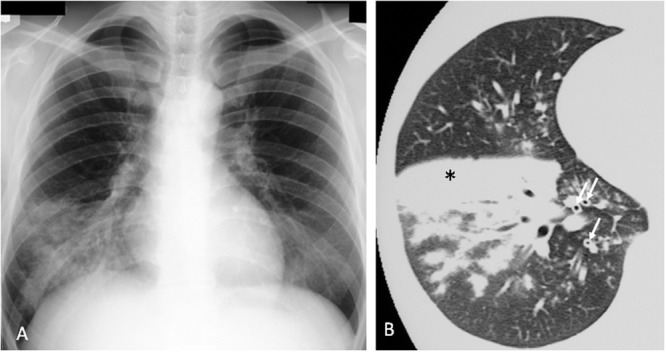
***Mycoplasma pneumoniae* pneumonia in human. (A)** Chest x-ray shows infiltrates in the right lower lobe. **(B)** Consolidation (^∗^) and bronchovascular bundles thickening (↑) on CT scan. Reproduced with permission from Tanaka and Hayashi (2007).

The most common radiographic findings consist of unilateral or bilateral areas of air-space consolidation and ground-glass opacities. However, the findings are variable and can include reticular or nodular opacities. Associated features include bronchial wall thickening and occasionally small pleural effusion. CT shows more accurately the presence and extent of centrilobular nodules, the lobular distribution of ground-glass opacities and a small amount of pleural effusion not visible on chest radiograph. Typical findings of adult *M. pneumoniae* pneumonia on chest radiograph and CT are demonstrated in **Figure 4**. The findings of bronchiolitis and lobular consolidation seen in histopathological specimens were seldom apparent on radiography but were commonly evident on CT. The most distinct abnormality seen on CT consisted of poorly defined centrilobular nodules, suggesting bronchiolitis. A study using high-resolution CT shows the most frequent chest radiologic finding was air-space consolidation, seen in 86% of 28 patients, and most commonly involving the lower lobe and nodular opacities were detected in 14 patients (Reittner et al., 2000). The areas of patchy air-space consolidation or ground-glass attenuation frequently had a lobular distribution, a characteristic pathological feature of bronchopneumonia. Although the most common abnormalities were thickening of the axial interstitium appearing bronchovascular bundles thickening on radiograph and CT (Tanaka et al., 1985).

## Pathological Findings in Open Lung Biopsy

**FIGURE 5:**
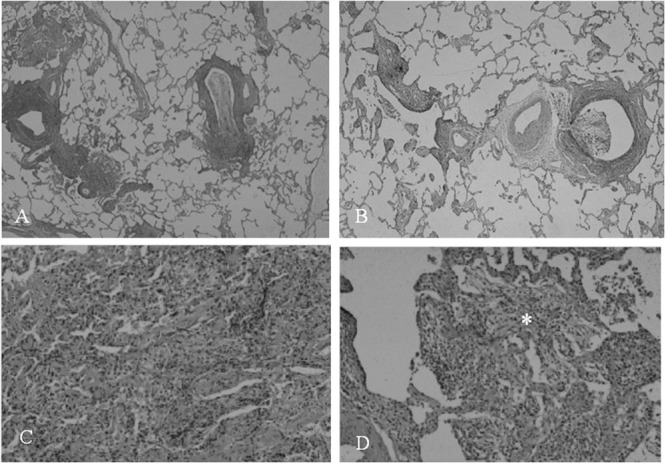
**Photomicrograph of open lung biopsy specimens in recovery phase of patients with *M. pneumoniae* pneumonia.** Low-magnification views of small airways show cellular bronchiolitis and exudate in the lumen **(A,B)**. High-magnification of alveolar area disclose stuffed alveoli with exudate, fibrin, neutrophil, and granulation tissue in alveolar duct (^∗^; **C,D**). Reproduced with permission from Tanaka (2016).

*Mycoplasma pneumoniae* organism selectively attaches airway ciliated epithelial cells (Tanaka et al., 2014) and therefore the pathological findings are usually limited to the airway walls as far down as small airways; the respiratory bronchioles. Histopathologically, *M. pneumoniae* pneumonia is characterized by acute cellular bronchiolitis with edematous and ulcerative lesions of bronchial walls and by peribronchial and perivascular interstitial opacities containing lymphocytes, plasma cells, and macrophages. The wall of bronchioles contains mononuclear cell and macrophage with a centrilobular distribution. In cases of severe pneumonia, diffuse alveolar damage with fibrinous exudates and hyaline membrane formation (Rollins et al., 1986; Izumikawa et al., 2014). Histopathological observation of open lung biopsy specimens from middle aged woman in recovery phase of *M. pneumoniae* pneumonia are showed in **Figure 5**. Low-magnification photomicrographs of small airways shows cellular bronchiolitis with thickening walls and exudates in the lumen. High-magnification of alveolar area discloses intra-alveolar inflammatory-cell infiltration and organizing pneumonia with granulation tissue filling alveolar ducts.

**FIGURE 6:**
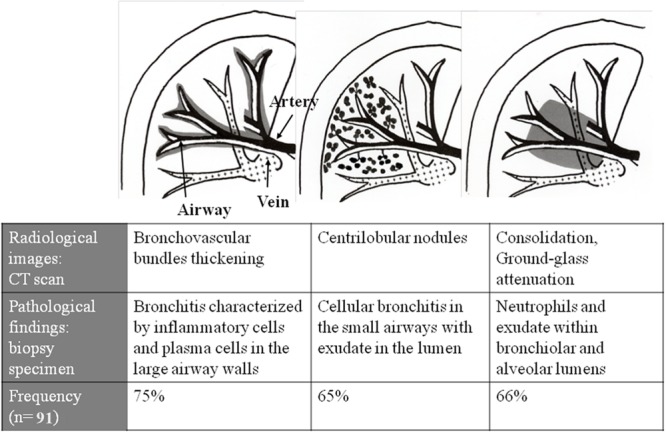
**Summary of radiological–pathological correlation in adult *M. pneumoniae* pneumonia.** Reproduced with permission from Tanaka et al. (1997).

**FIGURE 7:**
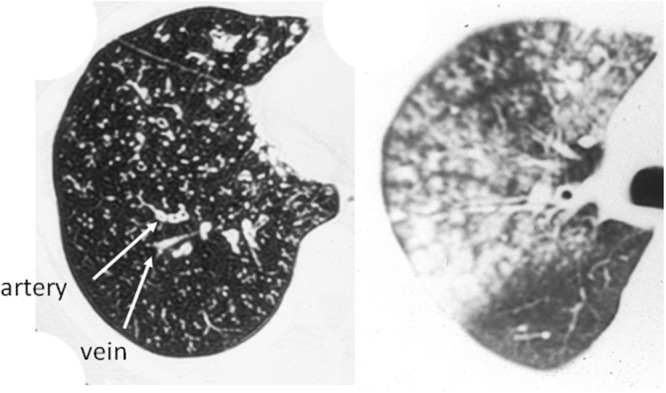
**Computed tomography of centrilobular nodules predominant pattern in two patients with *M. pneumoniae* pneumonia.** Reproduced with permission from Tanaka et al. (2004).

Summary of pathological–radiological correlations and frequency of three major CT findings in 91 cases of adult *M. pneumoniae* pneumonia were shown in **Figure 6**. It was reported that *M. pneumoniae* pneumonia in the recovery phase showed predominantly centrilobular nodular patterns, which disclosed immunological inflammation remaining in the small airways (Tanaka et al., 1985). However, some patients demonstrated centrilobular nodules in the early phase of the pneumonia (**Figure 7**), which mimicking T-helper type 1 pulmonary host response in the mice model (Tanaka et al., 1996a).

## Host CMI and Radiological Pattern

The CMI of the host plays an important role in the development of *M. pneumoniae* pneumonia. *M. pneumoniae* pneumonia in patients with immunodeficiency syndrome had a rack of radiological chest findings (Foy et al., 1973). On the other hand, the radiographic appearance of *M. pneumoniae* pneumonia in patients with sarcoidosis showed a bilateral reticulonodular pattern (Putman et al., 1975). Ito et al. (1995) reported a married couple who developed *M. pneumoniae* pneumonia at the same time, and whose severity of pneumonia, radiological findings and serum soluble IL-2 receptor levels were marked different between wife and husband. The wife developed acute respiratory failure with high serum levels of soluble IL-2 receptor, on the other hand, the husband suffered from pneumonia with a moderate elevation of soluble IL-2 receptor. The difference may be reflected in the serum soluble IL-2 receptor levels, a marker of T cell activation *in vivo*. Serum IL-18 levels in patients with severe *M. pneumoniae* pneumonia were higher than those in mild cases (Tanaka et al., 2002), which suggested IL-18 and T-helper 1(Th1) cytokines may play a significant role in developing pneumonia. And IL-18 levels of pleural effusion in pediatric patients also elevated (Narita et al., 2000). In human *M. pneumoniae* pneumonia, positive rate of purified protein derivative (PPD) test in patients with nodular opacities predominant pattern on CT (group N) was higher than that in patients with air-space consolidation or ground-glass opacities predominant pattern on CT (group C) in *M. pneumoniae* pneumonia (Tanaka et al., 1996b). The PPD skin reaction; tuberculin skin test, is used not only to confirm past infection of *Mycobacterium tuberculosis* but also to determine the CMI of the host. In other words, patients with nodular opacities predominant on CT showed a more marked response to PPD than those with air-space consolidation predominant pattern on CT.

## Bronchiolitis Obliterans Following *M. pneumoniae* Infection

**FIGURE 8:**
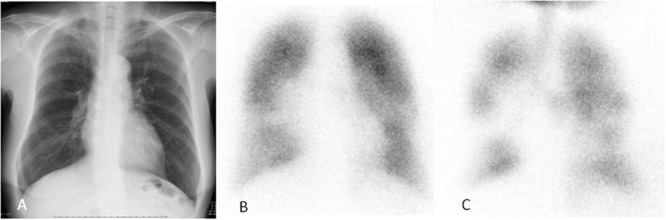
**Bronchiolitis obliterans following *M. pneumoniae* infection. (A)** Chest X-ray showing normal. **(B)** ^99m^Tc-MAA perfusion scan revealing slight defect. **(C)** ^81m^Kr-aerosol ventilation demonstrating prominent multiple defects throughout the lung field. Reproduced with permission from Tanaka (2016).

The presence of centrilobular nodules in a patchy distribution is characteristic of infectious bronchiolitis, allowing distinction from non-infectious causes of bronchiolitis, which usually have a diffuse distribution throughout both lungs (Chan et al., 1999; Ebnother et al., 2001). We experienced a woman suffered *M. pneumoniae* infection. She complained dyspnea 2 months after the infection. Her chest radiograph and CT revealed overinflation with no centriacinar nodules (**Figure 8**). Her pulmonary function test revealed a vital capacity of 2469 ml, an forced effort in 1 s; FEV1 of 940 ml, and FEV1/FVC ratio of 41%, a V50/V25 ratio of 2.01, and a residual volume/total lung capacity ratio of 47%. ^99m^Tc-MAA perfusion scan revealed slight defect and ^81m^Kr-aerosol ventilation scan demonstrated prominent multiple defects, suggesting bronchiolitis obliterans. Centrilobular nodule of under 500 μm could not detected in high-resolution CT scan, therefore pulmonary functions are useful technique for detecting subtle small airway abnormality.

## Author Contributions

The author confirms being the sole contributor of this work and approved it for publication.

## Conflict of Interest Statement

The author declares that the research was conducted in the absence of any commercial or financial relationships that could be construed as a potential conflict of interest.
